# Phylogenetic structure of European *Salmonella *Enteritidis outbreak correlates with national and international egg distribution network

**DOI:** 10.1099/mgen.0.000070

**Published:** 2016-08-25

**Authors:** Tim Dallman, Thomas Inns, Thibaut Jombart, Philip Ashton, Nicolas Loman, Carol Chatt, Ute Messelhaeusser, Wolfgang Rabsch, Sandra Simon, Sergejs Nikisins, Helen Bernard, Simon le Hello, Nathalie Jourdan da-Silva, Christian Kornschober, Joel Mossong, Peter Hawkey, Elizabeth de Pinna, Kathie Grant, Paul Cleary

**Affiliations:** ^1^​ Public Health England, UK; ^2^​ Department of Infectious Disease Epidemiology, Imperial College, London, UK; ^3^​ Institute of Microbiology and Infection, University of Birmingham, Birmingham, UK; ^4^​ Bavarian Health and Food Safety Authority, Oberschleissheim, Germany; ^5^​ Robert Koch Institute, Division for Enteropathogenic Bacteria and Legionella, Wernigerode, Germany; ^6^​ Department for Infectious Disease Epidemiology at the Robert Koch Institute, Berlin, Germany; ^7^​ Institut Pasteur, Centre national de reference des E. coli, Shigella et Salmonella, Paris, France; ^8^​ French Institute for Public Health Surveillance, France; ^9^​ Austrian Agency for Health and Food Safety, National Reference Centre for Salmonella, Graz, Austria; ^10^​ Surveillance & epidemiology of infectious diseases, Laboratoire National de Santé, Dudelange, Luxembourg

**Keywords:** Salmonella, whole-genome sequencing, traceback investigation, foodborne outbreak

## Abstract

Outbreaks of *Salmonella *Enteritidis have long been associated with contaminated poultry and eggs. In the summer of 2014 a large multi-national outbreak of *Salmonella *Enteritidis phage type 14b occurred with over 350 cases reported in the United Kingdom, Germany, Austria, France and Luxembourg. Egg supply network investigation and microbiological sampling identified the source to be a Bavarian egg producer. As part of the international investigation into the outbreak, over 400 isolates were sequenced including isolates from cases, implicated UK premises and eggs from the suspected source producer. We were able to show a clear statistical correlation between the topology of the UK egg distribution network and the phylogenetic network of outbreak isolates. This correlation can most plausibly be explained by different parts of the egg distribution network being supplied by eggs solely from independent premises of the Bavarian egg producer (Company X). Microbiological sampling from the source premises, traceback information and information on the interventions carried out at the egg production premises all supported this conclusion. The level of insight into the outbreak epidemiology provided by whole-genome sequencing (WGS) would not have been possible using traditional microbial typing methods.

## Data Summary

1. FASTQ sequences were deposited in the NCBI Short Read Archive under the BioProject PRJNA248792 (http://www.ncbi.nlm.nih.gov/bioproject/?term=PRJNA248792)
2. Supplementary material is available at the following git repository https://github.com/timdallman/sent_14b.git

## Impact Statement

In this article we show how the phylogenetic relationships between isolates in a foodborne outbreak can be informative in revealing underlying epidemiological trends.

We were able to show a clear statistical correlation between the topology of the UK cases, the egg distribution network and the phylogenetic network of outbreak isolates. This indicated that the phylogeny clustered into distinct clades related to the source of eggs.

This study shows the benefit of whole-genome sequencing of pathogens in revealing the true epidemiology behind an outbreak allowing inferences to be made about source diversity and food-chain contamination.

## Introduction

*Salmonella* Enteritidis outbreaks in humans are often linked to contaminated foodstuffs produced by the poultry industry (Harker *et al*., 2014; Lane *et al*., 2014). The incidence of *Salmonella* Enteritidis in the United Kingdom and in Europe has decreased significantly following the implementation of a vaccination program and other control measures in chicken flocks(Poirier *et al*., 2008), but outbreaks associated with contaminated eggs continue to occur (Hugas & Beloeil, 2014).

In 2014 a large multi-national outbreak of *Salmonella* Enteritidis phage type 14b was linked to consumption of eggs (Inns *et al*., 2015). Over 350 cases were reported in the United Kingdom, Germany, Austria, France and Luxembourg. Egg supply network investigation and microbiological sampling identified the source to be a Bavarian egg producer.

Whole-genome sequencing (WGS) is increasingly used for surveillance of food-borne pathogens (Dallman *et al.*, 2015b; Quick *et al.*, 2015; Jenkins *et al.*, 2015) and prospective typing of *Salmonella* Enteritidis isolates can identify possible outbreaks in real-time (den Bakker *et al.*, 2014; Deng *et al.*, 2014). *Salmonella* Enteritidis outbreaks can be investigated using WGS as part of the case definition, as strains of a given outbreak are typically monophyletic (i.e. representing a single evolutionary pathway), with limited diversity between outbreak isolates (Deng *et al.*, 2014; Wuyts *et al.*, 2015; Taylor *et al.*, 2015). WGS has however revealed significant polyclonal contamination within chicken production farms (Allard *et al.*, 2013).

Phylogenetic methods that explore the relationships between microbial genomes have been used to study the emergence (Dallman *et al.*, 2015a; Holt *et al.*, 2012), geographical diffusion (Baker *et al.*, 2015; He *et al.*, 2013) and transmission of infections (Bryant *et al.*, 2013; Eyre *et al.*, 2013). Phylogenetic topologies can also be informative in terms of source attribution in outbreak investigations (Harris *et al.*, 2013). In this study we explore the relationships between the observed phylogeny of 400 clinical, environmental and food isolates obtained in this outbreak and the distribution network of the implicated foodstuff, as ascertained by national and international traceback investigations.

## Methods

### Strains.

Since April 2014 all presumptive isolates of *Salmonella* in England and Wales received by the Gastrointestinal Bacteria Reference Unit at Public Health England (PHE) have undergone WGS. As of 1st August 2015, 3844 sequences of *Salmonella enterica* serovar Enteritidis had been analysed for routine surveillance purposes. A set of 44 isolates from Germany, France, Austria and Luxembourg were sequenced as part of this investigation with the inclusion criteria as follows; clinical isolates belonging to phage type 14b, clinical isolates with matching multi-locus variable number tandem repeat analysis (MLVA) profile 2-12-7-3-2, and implicated food or environmental samples. Of all the isolates sequenced from the UK and mainland Europe, 401 strains of *Salmonella* Enteritidis matched the outbreak single-nucleotide polymorphism (SNP) address 1.2.3.38.38.38 (Ashton *et al.*, 2015), these included all the isolates previously described by Inns **et al.** (2015). The strains are described in Table S1, available in the online version of this paper.

### Food chain investigations.

Rapid Alert System for Food and Feed (RASFF) notifications were issued on 09 July 2014 (France), 31 July 2014 (Austria) and 1 August 2014 (France), which linked *S.* Enteritidis outbreaks in France and Austria to chicken eggs from Company X in Germany. Company X had four separate premises, three in Germany and one in the Czech Republic; all are operationally independent. All four sites used young chickens (pullets) from two locations: one in Germany and one in the Czech Republic. Food supply network investigations involved obtaining information on the supply of eggs from Company X to UK distributors and tracing onward supply to other UK companies. In addition, supply network investigations were conducted in England to trace supplies of chicken and chicken eggs consumed by cases to their source as described in Inns **et al.** (2015). In total, 198 of the 287 (69 %) confirmed UK cases could be plausibly linked to eggs supplied by one company, Company X, with no traceback information available for the other cases.

### Sequencing.

Sequencing was performed by the PHE Genome Sequencing Unit using Nextera library preparation on a HiSeq 2500 (Illumina) run in fast mode according to the manufacturers’ instructions, which yielded 2×100 base pair paired-end reads. At the Robert Koch Institute, University of Birmingham and Institut Pasteur, libraries from genomic DNA were created using the Nextera library preparation kit and subsequently run on the Illumina MiSeq sequencer using Illumina’s v3 Reagent Kit to produce 2×300 base pair paired-end reads.

High-quality Illumina reads were mapped to the *Salmonella enterica* Enteritidis reference genome (GenBank:AM933172) using BWA-MEM (Li & Durbin, 2010). SNPs were then identified using GATK2 (McKenna *et al.*, 2010) in unified genotyper mode. Core genome positions that had a high quality SNP (>90 % consensus, minimum depth 10×, GQ >=30) in at least one strain were extracted and RaxML v8.17 (Stamatakis, 2014) used to derive the maximum-likelihood phylogeny of the isolates under the GTRCAT model of evolution. Single-linkage SNP clustering was performed as previously described (Ashton *et al.*, 2015). FASTQ reads from all sequences in this study can be found at the PHE Pathogens BioProject at the National Center for Biotechnology Information (Accession PRJNA248792).

### Timed phylogenies.

Timed phylogenies were reconstructed using BEAST-MCMC v1.80 (Drummond *et al.*, 2012) after first removing regions of the genome predicted to have undergone recombination using Gubbins v1.3 (Croucher *et al.*, 2015). Alternative clock models and population priors were computed and assessed based on Bayes Factor (BF) tests using Tracer v1.6. The highest supported model was a relaxed lognormal clock rate under a constant population size. All models were run with a chain length of one billion. A maximum clade credibility tree was constructed using TreeAnnotator v1.75 (Drummond *et al.*, 2012).

### Network comparison.

Distances on the distribution network were measured as the number of intermediate nodes on the shortest path between a pair of cases. For instance, the distance between two cases infected in the same restaurant was one. The genetic distance between isolates of two different cases was measured as the patristic distances between the corresponding tips of the phylogeny. A Monte Carlo Mantel test (Mantel, 1967) was used to investigate the degree of correlation between the supply network and genetic distance matrices, using 167 cases that were both sequenced and documented on the distribution network, with 9999 random permutations of the data. Because the relationship may be driven by cases infected by the same source, we also tested correlations between distances based on pairs of cases with different sources of infection only.

In addition, we accounted for the potential bias stemming from the existence of distinct genetic clades in the sampled isolates using a partial Mantel test (Legendre & Legendre, 2012). In this analysis, a linear regression is used to predict patristic distances as a function of a binary clade membership distance (0=same clade; 1=different clades) and distances on the food distribution network. Effects of each covariate and their interaction were tested using the classical ANOVA framework (Legendre & Legendre, 2012).

All analyses were carried out in the R software (Team, 2014), using the packages igraph (Csardi & Nepusz, 2006) for graph distances, adephylo (Jombart *et al.*, 2010) for phylogenetic distances computations and ade4(Dray *et al.*, 2007, p. 4) for the Mantel test.

## Results

**Fig. 1.**
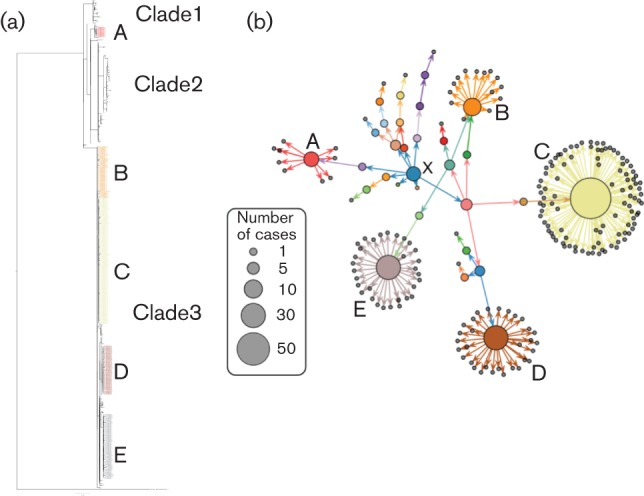
(a) Maximum-likelihood phylogeny based on whole-genome sequences of 401 isolates implicated in the outbreak rooted against an unrelated isolate of *S.* Enteritidis isolated from Luxembourg. (b) Distribution network for the 167 cases that were both sequenced and documented on the network, with arrows representing likely contaminations. Black circles represent cases, while internal nodes (sources) are represented as coloured disks, with a size proportional to the number of subsequent infections. The five point-source outbreaks associated with three Chinese restaurants (B, C, D), a hospital (E) and kebab grill (A) are coloured on the phylogeny and labelled on the trace-back network. Company X is the blue centroid sphere.

The phylogeny of 401 isolates implicated in the outbreak resolved into three clades all supported with bootstraps >95 (Fig. 1a). The isolates formed a single five-SNP single-linkage cluster with a maximum distance between any two genomes of 23 SNPs. Within England, the outbreak consisted of five point-source, geographically distinct incidents and 101 sporadic cases. Isolates from the point-source outbreaks resolved into distinct sub-clades in the outbreak phylogeny with a maximum SNP distance of 2 (mode of 0). Supply network information plausibly linked 198 cases in England to eggs supplied by one company, Company X (Fig. 1b). Multiple trace-back pathways to the implicated source of infection were identified (Inns *et al.*, 2015).

**Fig. 2.**
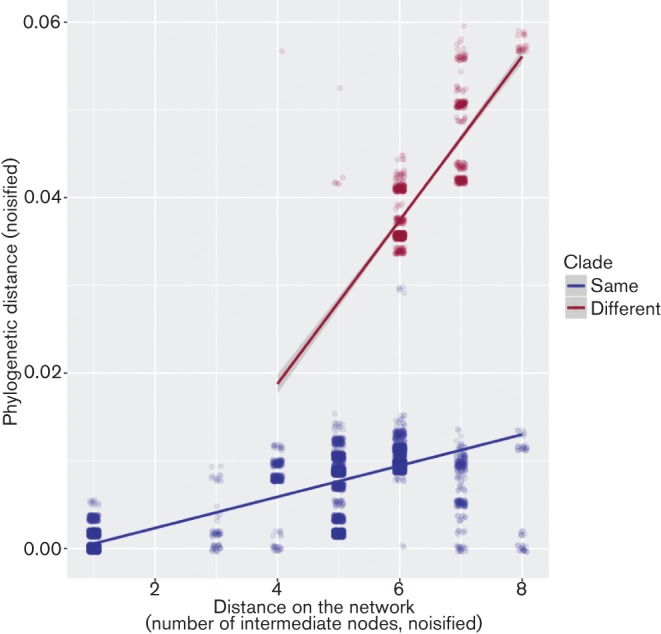
Scatterplot showing the relationship between the phylogenetic distance and the distance between cases on the traceback network, after accounting for the existence of two distinct genetic clades in sampled isolates. Each dot represents a pairwise comparison between two cases. Data have been slightly noisified to better visualise overlapping points. Dots are colored according to clade memberships, with pairs of isolates from the same clade in blue, and from different clades in red. Lines indicate predictions of a linear model using different slopes for each group.

According to the Monte-Carlo Mantel test, genetic distances between isolates significantly increased with the distance (number of intermediate nodes) on the distribution network (*r*=0.62, *P*=0.0001). This relationship remained significant when considering only pairs of cases from different direct exposures (*r*=0.46; *t*-test: *P*=2.2×10^−16^) and was robust to non-linearities between distances (Spearman ρ=0.60, *P*=2.2×10^−16^). This relationship also remained significant when accounting for the existence of distinct genetic clades (ANOVA: *F*=18,023; *P*<2×10^−16^; Fig. 2). In fact, most of the variance in patristic distances could be explained by clade membership and by the accumulation of mutations during the outbreak (R^2^=0.94; *P*<2×10^−16^), with 0.18 % [CI_95 %_: (0.17 %; 0.18 %)] genome diversity accumulated on average between nodes of the food network within a given clade (Fig. 2).

The outbreak occurred over a period of 17 weeks in England and Wales. To test the hypothesis that this length of time was insufficient for the observed level of nucleotide diversity to occur during the outbreak period two timed phylogenies were constructed. Firstly a mutation rate of 3.4 [95 % highest posterior density (HPD) 2.6–5.1] mutations per genome per year was estimated based on 142 diverse (50 SNP cluster) representatives of the *Salmonella* Enteritidis PHE collection. This mutation rate is three times faster than that predicted by Deng *et al.* (2014), although the predicted time to most recent common ancestor of the lineages is consistent, suggesting that the differences lie in the SNP-calling algorithms. Secondly a mutation rate of 8.35 (95 % HPD 5.8–11.0) mutations per genome per year was estimated based on the outbreak isolates only. The faster short-term mutation rate between *Salmonella* outbreak samples has previously been described (Hawkey *et al.*, 2013) and maybe due to intense sampling of minority variants pre-fixation. Regardless of these discrepancies no analysis predicts a mutation rate that provides sufficient time for accumulation of the diversity observed during this international outbreak. The time to the most recent common ancestor for the three clades was estimated to be 2.9 years (95 % HPD 2.5–3.2 years) (Fig. S1). The correlation between phylogenetics and the egg distribution network could however be explained by either a single sampling event from a single diverse source into different parts of the food network or by multiple sampling from compartmentalised diversity in the source population into different parts of the food network.

**Fig. 3.**
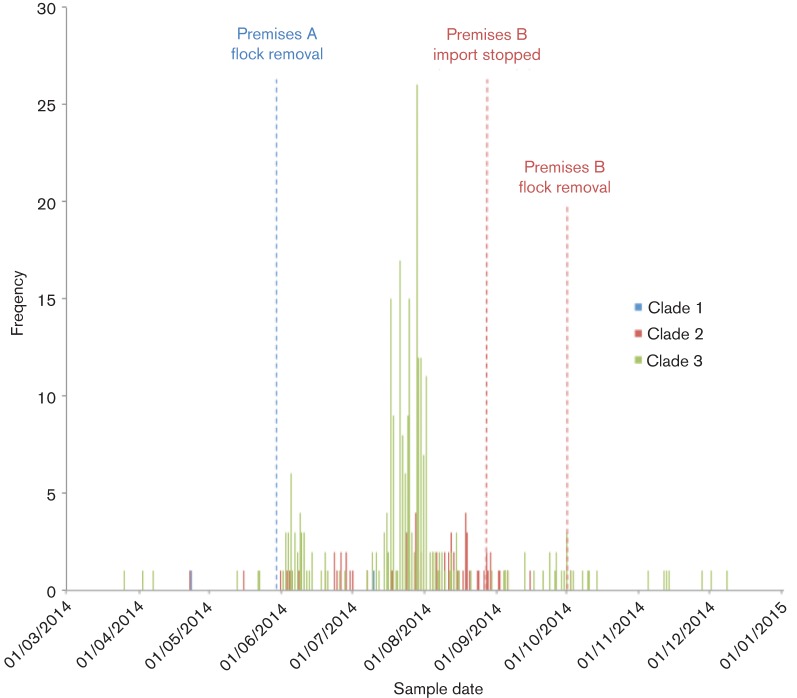
The epidemic curve of English cases over nine months with the interventions at sites of company X indicated by vertical lines. Cases are coloured by phylogenetic clade.

A set of 44 isolates from countries other than England were sequenced, including isolates from clinical cases, implicated foodstuffs and both environmental sampling and eggs from the implicated egg producer (Table 1). Twelve sequences from isolates directly sampled from premises A, or from eggs that could be directly linked by batch number to premises A of company X clustered unilaterally into clade 1 of the phylogeny (Fig. 1a). Clade 1 included three clinical cases from Germany and three cases from England. Two further clinical isolates from France in clade 1 were from cases where trace-back information linked them to eggs from premises A of company X. Two egg isolates from France could be traced to premises A due to their egg-mark. Environmental sampling from premises A yielded six isolates of *S.* Enteritidis from eggs, three from poultry and one from the production environment; all clustered into clade 1. After the link with clinical cases in France was identified, premises A was deep cleaned following culling of the flock (Fig. 3).

**Table 1.** Strain list of samples from outside the United Kingdom Traceback informtion relates to premises A or B from company X. ?, No traceback information available.

Strain	Country	Clade	Traceback	Source
H143980751	Germany	1	A	Egg
H143980752	Germany	1	A	Egg
H143980753	Germany	1	A	Egg
H143980754	Germany	1	A	Egg
H143980755	Germany	1	A	Egg
H143980756	Germany	1	A	Egg
H143360569	France	1	A	Human
H143360570	France	1	A	Egg
H143360571	France	1	A	Egg
201405122	France	1	A	Human
14-06145	Germany	1	?	Human
14-05226	Germany	1	A	Environmental
14-05225	Germany	1	A	Poultry
14-05224	Germany	1	A	Poultry
14-05227	Germany	1	A	Poultry
14-06012	Germany	1	?	Human
14-06175	Germany	1	?	Human
H143720773	Luxembourg	2	?	Human
H143980750	Germany	2	B	Egg
H143980757	Germany	2	B	Egg
H143380471	Austria	2	?	Human
H143380472	Austria	2	?	Human
H143380473	Austria	2	?	Human
H143380474	Austria	2	?	Human
H143360568	France	2	?	Human
201405861	France	2	?	Human
201405760	France	2	B	Human
201405756	France	2	B	Human
201405757	France	2	B	Human
14-04296	Germany	2	?	Human
14-04310	Germany	2	?	Human
14-04552	Germany	2	?	Human
14-04639	Germany	2	?	Human
14-04870	Germany	2	?	Human
14-05946	Germany	2	?	Human
14-06388	Germany	2	?	Human
H143380470	Austria	3	?	Human
H143380475	Austria	3	?	Human
14-05567	Germany	3	?	Human
14-05569	Germany	3	?	Human
14-05795	Germany	3	?	Human
14-05568	Germany	3	?	Human
H143360566	France	Non-outbreak	?	Human
H143360567	France	Non-outbreak	?	Human
14-06148	Germany	Non-outbreak	?	Human

Two isolates from eggs sampled at premises B of company X clustered in clade 2. Clade 2 included five clinical cases from France, of which three were linked to eggs from premises B of company X, as well as seven clinical cases from Germany, four from Austria, one from Luxembourg and 32 from England. Nine of the English cases were linked to a point source outbreak at a kebab restaurant.

Clade 3 included the majority of isolates from England, including isolates from four separate point source outbreaks. The clade contains four isolates from clinical cases in Germany and two isolates from clinical cases in Austria. Although cases were linked through exposure traceback to company X, no isolates from clade 3 were detected at premises A or B.

Fig. 3 shows the distribution of cases over time coloured by clade with interventions involving flock destruction and deep cleaning at premises A and B also depicted. The intervention at premises A preceded the majority of cases. Cases in Clade 3 continued after the interventions at premises A and B. The timeline and epidemiological data are congruent with the phylogenetic analysis. Isolates from clade 1 were not detected after interventions at premises A of company X (removal of the flock and cessation of egg delivery). Clade 2 strains were rarely detected from 26 days (estimated shelf life for eggs) after interventions at premises B of company X (cessation of egg delivery to the UK). Clade 3 strains, which were detected neither at premises A nor at premises B of company X, continued to be detected from May to November 2014 after interventions at premises A and B, but detections largely ceased from 26 days after the cessation of egg deliveries from the Czech premises of company X.

## Conclusion

Whole-genome sequencing allows the identification of linked cases of infection with unprecedented resolution. Due to the sequential nature of mutational drift, phylogenetic methods can be used to study variation in genomes and reveal ancestral relationships. The topology of such a phylogeny in the case of foodborne disease may reveal information about source diversity and how that diversity was sampled. In this study, sequences from a large outbreak of *S.* Enteritidis linked to the consumption of eggs originating from a Bavarian producer revealed a phylogeny with three clades forming a monophyletic cluster within the *S.* Enteritidis population. Isolates from five point-source outbreaks clustered themselves into distinct monophyletic clusters with minimal variation.

Traceback information in France led to sampling of two premises of Company X in Bavaria. Eggs, environmental samples and isolates from cases linked to premises A clustered phylogenetically into Clade 1 whereas eggs, environmental samples and isolates from cases linked to premises B clustered phylogenetically into Clade 2. Although the majority of cases in Clade 3 were linked via the supply network to Company X, clade 3 strains were not identified in samples from premises A or B. Interventions including flock destruction and disinfection at premises A and premises B coincided with a cessation of cases from Clades 1 and 2. Cases in clade 3 continued sporadically for several months after these interventions, however the frequency decreased following the import suspension of eggs from the Czech premises of company X. We conclude that the origin of Clade 3 is most likely to be another location within company X or its suppliers. The three clades’ common ancestor existed approximately three years previously.

The supply network investigation for English cases revealed a complex egg distribution network consisting of several distinct distribution chains. We were able to show a clear statistical correlation between the topology of the UK egg distribution network and the phylogenetic network of outbreak isolates. This correlation can most plausibly be explained by different parts the egg distribution network being supplied by eggs solely from independent premises of Company X. This resulted in a phylogeny that clusters into distinct clades related to the source of eggs. When accounting for these clades, almost all of the genetic variation could be explained by the process of diffusion of isolates over the food distribution network (R^2^=0.94; P<2×10^−16^).

In this paper, we used a simple approach to quantify the extent of the association between the phylogeny of sampled isolates and the food distribution network, which is only partially known. Our approach captures the topology of the food distribution network using distances between cases, computed as the shortest path between the corresponding nodes. While crude, this measure should be robust to the addition of nodes and edges, as long as the closest food providers linking the cases have been reported. Therefore, our results should remain identical when considering the full food distribution network.

This is the first time, to our knowledge, that phylogenetic data have been combined with food supply network data in the context of an infectious disease outbreak investigation. Our results suggest that combining whole-genome sequencing with information on the food distribution network permits a more detailed exploration of possible sources of infection in outbreak situations and to inform interventions. We recommend that further work be undertaken to develop and standardise the methods used to compare phylogenetic and food supply network information, to enable use of these techniques in future outbreaks to help identify sources and guide the implementation of public health control measures to prevent further illness.

The level of insight into the outbreak epidemiology provided by WGS would not have been possible using traditional microbial typing methods routinely employed for *Salmonella* outbreak investigation such as MLVA, PFGE and phage typing. WGS provided the high-resolution typing needed that allowed the effectiveness of interventions at premises A and B to be observed. Similarly the robust, high discrimination of WGS provided the evidence for driving traceback in specific directions, which is of particular importance in complex foodborne source identification investigations. Finally the digital nature of WGS data allowed data to be readily exchanged and analysed between four institutions in different countries.

## Supplementary Data

165 Supplementary File 1
