# Supplementary material for: Phylogenetic structure of European Salmonella Enteritidis outbreak correlates with national and international egg distribution network
Source: Microb Genom. 2016 Aug 25;2(8):e000070. doi: 10.1099/mgen.0.000070 (PMC5320589; doi:10.1099/mgen.0.000070)
Supplement: Supplementary file 1 [file mgen-02-70-s001.pdf]

ID	SRA accession	BioSample accession	Source	Country	Sample Date	SNP Adderss (t250.t100.t50.t25.t10.t5.t0)
9286	SRR1966195	SAMN03476279	Human	England	01.05.2014	1.1.1.1.1.1.1
54060	SRR1958325	SAMN03466218	Human	England	25.09.2014	1.1.101.174.174.174.174
70666	SRR1963269	SAMN03469728	Human	England	27.11.2014	1.1.105.178.254.254.254
63914	SRR1967019	SAMN03477097	Human	England	05.11.2014	1.1.119.217.217.217.217
63932	SRR1969284	SAMN03479361	Human	England	06.11.2014	1.1.122.221.221.221.221
65019	SRR1966723	SAMN03476801	Human	England	04.11.2014	1.1.124.226.226.226.226
65407	SRR1967973	SAMN03478064	Human	England	17.09.2014	1.1.127.233.233.233.233
67566	SRR1965713	SAMN03475821	Human	England	11.11.2014	1.1.130.237.237.237.237
68689	SRR1968780	SAMN03478878	Human	England	12.11.2014	1.1.131.239.239.239.239
68692	SRR1965912	SAMN03476004	Human	England	12.11.2014	1.1.134.242.242.242.242
68696	SRR1969258	SAMN03479335	Human	England	13.11.2014	1.1.135.243.243.243.243
68894	SRR1959468	SAMN03468648	Human	England	23.10.2014	1.1.136.244.244.244.244
69702	SRR1960378	SAMN03469111	Human	England	19.11.2014	1.1.138.247.247.247.247
73223	SRR1967006	SAMN03477084	Human	England	10.12.2014	1.1.147.263.263.263.263
73535	SRR1961505	SAMN03469160	Human	England	19.09.2014	1.1.148.264.264.264.264
73554	SRR1963454	SAMN03469900	Human	England	22.09.2014	1.1.150.266.266.266.266
73611	SRR1959386	SAMN03468543	Human	England	09.12.2014	1.1.151.268.268.268.268
6446	SRR1969592	SAMN03479668	Human	England	17.04.2014	1.1.156.275.275.275.275
7410	SRR1967781	SAMN03477871	Human	England	29.04.2014	1.1.157.276.276.276.276
H120540611	SRR1646243	SAMN03169583	Human	England	01.02.2012	1.1.162.286.286.286.286
H122420304	SRR1646133	SAMN04575166	Human	England	07.06.2012	1.1.168.297.297.297.297
H123740519	SRR1645445	SAMN03168805	Human	England	03.09.2012	1.1.176.315.315.315.812
H123580521	SRR1645782	SAMN03169047	Human	England	22.08.2012	1.1.177.321.321.321.321
H124680351	SRR1635097	SAMN03152399	Human	England	07.11.2012	1.1.185.343.343.343.343
29960	SRR3049245	SAMN04363532	Human	England	23.07.2014	1.1.191.358.363.364.366
83588	SRR1966609	SAMN03476690	Human	England	27.01.2015	1.1.198.484.603.644.780
133214	SRR3285283	SAMN04574820	Human	England	14.07.2015	1.1.2.1066.1749.2054.3119

13835	SRR1968807	SAMN03478905	Human	England	15.05.2014	1.1.2.1074.1763.2074.3174
45610	SRR1969527	SAMN03479604	Human	England	09.09.2014	1.1.2.137.137.137.137
48243	SRR1958152	SAMN03466052	Human	England	15.09.2014	1.1.2.149.149.149.149
51262	SRR1966595	SAMN03476676	Human	England	17.09.2014	1.1.2.167.167.167.167
53129	SRR1969657	SAMN03479733	Human	England	29.09.2014	1.1.2.173.173.173.173
206728	SRR3285244	SAMN04574798	Human	England	12.01.2016	1.1.2.197.544.2403.4325
68690	SRR1967133	SAMN03477223	Human	England	12.11.2014	1.1.2.240.240.240.240
13360	SRR1965552	SAMN03475674	Human	England	15.04.2014	1.1.2.360.368.369.3150
50150	SRR1958578	SAMN03466471	Human	England	24.09.2014	1.1.2.43.43.380.2290
85758	SRR1969589	SAMN03479665	Human	England	09.02.2015	1.1.200.491.619.660.802
80307	SRR1959222	SAMN03468425	Human	England	13.01.2015	1.1.202.526.680.735.915
99068	SRR1965636	SAMN03475749	Human	England	27.03.2015	1.1.204.580.786.862.1101
96239	SRR1965938	SAMN03476029	Human	England	16.03.2015	1.1.208.623.859.955.1226
95017	SRR1968045	SAMN03478136	Human	England	11.03.2015	1.1.212.724.1061.1193.1591
78695	SRR1960166	SAMN03468975	Human	England	24.12.2014	1.1.222.1001.1587.1831.2556
110976	SRR3585376	SAMN05173227	Human	England	11.05.2015	1.1.228.1033.1681.1946.2785
183662	SRR3285109	SAMN04574794	Human	England	27.10.2015	1.1.228.1033.1681.1946.4002
18531	SRR3049367	SAMN04363661	Human	England	04.06.2014	1.1.229.1034.1684.1954.2823
119502	SRR3286886	SAMN04576529	Human	England	02.06.2015	1.1.233.1044.1704.1976.2889
120575	SRR3286870	SAMN04576520	Human	England	30.04.2015	1.1.234.1045.1708.1982.2900
122682	SRR3286836	SAMN04576497	Human	England	12.06.2015	1.1.236.1049.1714.1998.2934
122699	SRR3286863	SAMN04576516	Human	England	15.06.2015	1.1.237.1051.1716.2001.2940
134034	SRR3585352	SAMN05173207	Human	England	08.07.2015	1.1.242.1065.1746.2051.3115
185901	SRR3284689	SAMN04574151	Human	England	03.11.2015	1.1.242.1065.1746.2051.3585
134013	SRR3284736	SAMN04574688	Human	England	15.07.2015	1.1.245.1070.1756.2063.3141
143553	SRR3585347	SAMN05173203	Human	England	10.08.2015	1.1.250.1085.1794.2122.3293
152413	SRR3585348	SAMN05173204	Human	England	25.08.2015	1.1.256.1101.1827.2165.3375
20903	SRR3049061	SAMN04363295	Human	England	05.06.2014	1.1.26.31.31.31.31

179844	SRR3585355	SAMN05173209	Human	England	23.10.2015	1.1.277.1177.1995.2391.3912
185894	SRR3585367	SAMN05173224	Human	England	11.11.2015	1.1.280.1189.2017.2431.4023
38415	SRR1967310	SAMN03477400	Human	England	06.08.2014	1.1.30.99.99.99.99
31651	SRR1968212	SAMN03478303	Human	England	15.07.2014	1.1.34.74.74.74.74
23508	SRR1968511	SAMN03478604	Human	England	25.06.2014	1.1.38.47.47.47.47
9464	SRR1966205	SAMN03476289	Human	England	29.04.2014	1.1.4.9.9.9.9
27973	SRR1968306	SAMN03478397	Human	England	17.07.2014	1.1.44.59.59.59.59
197074	SRR3284773	SAMN04574724	Human	England	04.12.2015	1.1.47.214.1658.2500.4224
32392	SRR1970131	SAMN03480207	Human	England	25.07.2014	1.1.52.75.75.75.75
32527	SRR1965330	SAMN03475454	Human	England	23.07.2014	1.1.53.76.76.76.76
38412	SRR1968202	SAMN03478293	Human	England	06.08.2014	1.1.63.97.97.97.97
46122	SRR1958664	SAMN03466557	Human	England	29.08.2014	1.1.87.142.142.142.142
46128	SRR1958164	SAMN03466064	Human	England	29.08.2014	1.1.88.143.143.143.143
53076	SRR3049354	SAMN04363648	Human	England	30.09.2014	1.1.98.171.171.171.171
53113	SRR1969841	SAMN03479918	Human	England	29.09.2014	1.1.99.172.172.172.172
21065	SRR3049778	SAMN04364029	Human	England	03.06.2014	1.10.28.33.33.33.33
H123980391	SRR1646278	SAMN03169618	Human	England	17.09.2012	1.11.181.335.335.335.335
21091	SRR3048975	SAMN04363215	Human	England	03.06.2014	1.11.31.37.37.37.37
39508	SRR1968406	SAMN03478497	Human	England	18.08.2014	1.11.48.106.106.106.106
39476	SRR1969727	SAMN03479803	Human	England	15.08.2014	1.11.67.104.104.104.104
45579	SRR1968089	SAMN03478180	Human	England	09.09.2014	1.11.82.135.135.135.135
34191	SRR1968743	SAMN03478841	Human	England	29.07.2014	1.12.55.79.79.79.79
38414	SRR1966926	SAMN03477004	Human	England	06.08.2014	1.12.64.98.98.98.98
41987	SRR1965492	SAMN03475612	Human	England	27.08.2014	1.15.61.118.118.118.118
160806	SRR3585360	SAMN05173219	Human	England	10.09.2015	1.2.262.1118.1850.2199.3462
180813	SRR3285462	SAMN04575140	Human	England	10.09.2015	1.2.262.1137.1888.2254.3602
166245	SRR3284721	SAMN04574534	Human	England	23.09.2015	1.2.270.1145.1898.2264.3626
13413	SRR1969390	SAMN03479467	Human	England	16.05.2014	1.2.3.151.151.151.3167

9182	SRR1967526	SAMN03477616	Human	England	07.05.2014	1.2.3.18.278.278.278
38425	SRR1965692	SAMN03475801	Human	England	29.07.2014	1.2.3.38.38.38.1072
201405122	SRR3410217	SAMN04883174	Human	France	2014	1.2.3.38.38.38.1098
37037	SRR1968785	SAMN03478883	Food	France	2014	1.2.3.38.38.38.1098
9245	SRR1965948	SAMN03476039	Human	England	23.04.2014	1.2.3.38.38.38.1098
49691	SRR3417497	SAMN04884709	Human	Austria	2014	1.2.3.38.38.38.1111
23473	SRR1966582	SAMN03476664	Human	England	13.06.2014	1.2.3.38.38.38.1116
37835	SRR1966076	SAMN03476162	Human	England	28.07.2014	1.2.3.38.38.38.1127
62556	SRR1958614	SAMN03466506	Human	England	29.08.2014	1.2.3.38.38.38.1151
43936	SRR3049187	SAMN04363428	Human	England	21.08.2014	1.2.3.38.38.38.1151
40273	SRR1968309	SAMN03478400	Human	England	12.08.2014	1.2.3.38.38.38.1151
34198	SRR1967053	SAMN03477131	Human	England	24.07.2014	1.2.3.38.38.38.1153
40269	SRR1965742	SAMN03475846	Human	England	09.08.2014	1.2.3.38.38.38.1162
37806	SRR1970129	SAMN03480205	Human	England	30.07.2014	1.2.3.38.38.38.1162
24053	SRR1965483	SAMN03475604	Human	England	19.06.2014	1.2.3.38.38.38.1166
39486	SRR1966020	SAMN03476106	Human	England	04.08.2014	1.2.3.38.38.38.1184
37062	SRR1965730	SAMN03475836	Human	England	06.08.2014	1.2.3.38.38.38.1201
54053	SRR3417507	SAMN04884719	Food	Germany	2014	1.2.3.38.38.38.1255
53996	SRR3417502	SAMN04884714	Food	Germany	2014	1.2.3.38.38.38.1255
38718	SRR1967510	SAMN03477600	Human	England	07.08.2014	1.2.3.38.38.38.1288
23082	SRR1969517	SAMN03479594	Human	England	08.06.2014	1.2.3.38.38.38.1315
43925	SRR3049494	SAMN04363789	Human	England	13.08.2014	1.2.3.38.38.38.1351
57111	SRR1968319	SAMN03478410	Enviromental	England	01.08.2014	1.2.3.38.38.38.1365
38349	SRR1970133	SAMN03480209	Human	England	27.07.2014	1.2.3.38.38.38.1378
53973	SRR3417501	SAMN04884713	Food	Germany	2014	1.2.3.38.38.38.1394
62582	SRR1957842	SAMN03465744	Human	England	02.09.2014	1.2.3.38.38.38.1404
48189	SRR1957842	SAMN03465744	Human	England	02.09.2014	1.2.3.38.38.38.1404
45270	SRR1957842	SAMN03465744	Human	England	02.09.2014	1.2.3.38.38.38.1404

58576	SRR3049014	SAMN04363250	Human	England	28.09.2014	1.2.3.38.38.38.1418
37111	SRR1966745	SAMN03476823	Human	England	06.08.2014	1.2.3.38.38.38.1431
54047	SRR3417506	SAMN04884718	Food	Germany	2014	1.2.3.38.38.38.1438
21113	SRR3049901	SAMN04364147	Human	England	04.06.2014	1.2.3.38.38.38.1449
201405757	SRR3410219	SAMN04883177	Human	France	2014	1.2.3.38.38.38.1456
201405756	SRR3410218	SAMN04883175	Human	France	2014	1.2.3.38.38.38.1457
44683	SRR1957774	SAMN03465669	Human	England	29.08.2014	1.2.3.38.38.38.1469
42171	SRR1968063	SAMN03478154	Human	England	26.08.2014	1.2.3.38.38.38.1544
54038	SRR3417505	SAMN04884717	Food	Germany	2014	1.2.3.38.38.38.1555
54014	SRR3417504	SAMN04884716	Food	Germany	2014	1.2.3.38.38.38.1555
34204	SRR1969924	SAMN03480001	Human	England	28.07.2014	1.2.3.38.38.38.1595
46593	SRR1969365	SAMN03479442	Human	England	15.07.2014	1.2.3.38.38.38.1608
37045	SRR3417493	SAMN04884705	Human	Austria	2014	1.2.3.38.38.38.1757
21116	SRR3049304	SAMN04363598	Human	England	04.06.2014	1.2.3.38.38.38.1775
43930	SRR3049134	SAMN04363371	Human	England	14.08.2014	1.2.3.38.38.38.1795
38332	SRR1967887	SAMN03477978	Human	England	25.07.2014	1.2.3.38.38.38.1809
31975	SRR1968475	SAMN03478568	Human	England	25.07.2014	1.2.3.38.38.38.1903
60382	SRR1958504	SAMN03466397	Human	England	07.10.2014	1.2.3.38.38.38.2034
32475	SRR1969708	SAMN03479784	Food	England	17.07.2014	1.2.3.38.38.38.2060
20926	SRR3049503	SAMN04363796	Human	England	07.06.2014	1.2.3.38.38.38.2103
14-05226	SRR3410210	SAMN04883165	Enviromental	Germany	2014	1.2.3.38.38.38.2115
73134	SRR1966628	SAMN03476707	Human	England	01.12.2014	1.2.3.38.38.38.2129
49751	SRR1957820	SAMN03465716	Human	England	15.09.2014	1.2.3.38.38.38.2163
56991	SRR3049068	SAMN04363303	Human	England	30.09.2014	1.2.3.38.38.38.2244
34171	SRR1966928	SAMN03477006	Human	England	24.07.2014	1.2.3.38.38.38.2245
65448	SRR1965411	SAMN03475533	Human	England	03.10.2014	1.2.3.38.38.38.2246
41984	SRR1968882	SAMN03478980	Human	England	14.08.2014	1.2.3.38.38.38.2248
37038	SRR1969386	SAMN03479463	Food	France	2014	1.2.3.38.38.38.2314

36531	SRR1967772	SAMN03477862	Human	England	29.07.2014	1.2.3.38.38.38.2321
43884	SRR3049125	SAMN04363362	Human	England	13.08.2014	1.2.3.38.38.38.2366
37832	SRR1965506	SAMN03475628	Human	England	31.07.2014	1.2.3.38.38.38.2451
21119	SRR3049092	SAMN04363328	Human	England	04.06.2014	1.2.3.38.38.38.2483
37807	SRR1967170	SAMN03477260	Human	Wales	28.07.2014	1.2.3.38.38.38.2542
36522	SRR1965772	SAMN03475873	Human	England	23.07.2014	1.2.3.38.38.38.2621
54059	SRR3417508	SAMN04884720	Food	Germany	2014	1.2.3.38.38.38.2623
68972	SRR1966536	SAMN03476618	Human	England	11.11.2014	1.2.3.38.38.38.2646
21868	SRR3049229	SAMN04363515	Human	England	11.06.2014	1.2.3.38.38.38.2660
21132	SRR3048999	SAMN04363239	Human	England	31.05.2014	1.2.3.38.38.38.2669
14-06388	SRR3410216	SAMN04883172	Human	Germany	2014	1.2.3.38.38.38.2797
14-06012	SRR3410214	SAMN04883170	Human	Germany	2014	1.2.3.38.38.38.2798
14-05946	SRR3410213	SAMN04883169	Human	Germany	2014	1.2.3.38.38.38.2799
14-06175	SRR3410215	SAMN04883171	Human	Germany	2014	1.2.3.38.38.38.2800
14-05795	SRR3410212	SAMN04883168	Human	Germany	2014	1.2.3.38.38.38.2801
56112	SRR1966625	SAMN03476704	Human	England	12.09.2014	1.2.3.38.38.38.354
53110	SRR1965275	SAMN03475400	Human	England	23.09.2014	1.2.3.38.38.38.354
49693	SRR3417499	SAMN04884711	Human	Austria	2014	1.2.3.38.38.38.357
49692	SRR3417498	SAMN04884710	Human	Austria	2014	1.2.3.38.38.38.357
37046	SRR3417494	SAMN04884706	Human	Austria	2014	1.2.3.38.38.38.357
57623	SRR1958052	SAMN03465953	Human	England	08.08.2014	1.2.3.38.38.38.367
57102	SRR1965114	SAMN03474010	Human	England	11.08.2014	1.2.3.38.38.38.367
48262	SRR1957888	SAMN03465790	Human	England	05.09.2014	1.2.3.38.38.38.367
46591	SRR1967039	SAMN03477117	Human	England	18.07.2014	1.2.3.38.38.38.367
46590	SRR1968332	SAMN03478423	Human	England	17.07.2014	1.2.3.38.38.38.367
46589	SRR1967600	SAMN03477690	Human	England	17.07.2014	1.2.3.38.38.38.367
46581	SRR1970321	SAMN03480397	Human	England	18.07.2014	1.2.3.38.38.38.367
46580	SRR1966225	SAMN03476308	Human	England	17.07.2014	1.2.3.38.38.38.367

46579	SRR1968486	SAMN03478579	Human	England	17.07.2014	1.2.3.38.38.38.367
46011	SRR1968755	SAMN03478853	Human	England	09.08.2014	1.2.3.38.38.38.367
40799	SRR1965168	SAMN03474062	Human	England	15.08.2014	1.2.3.38.38.38.367
39506	SRR1969515	SAMN03479592	Human	England	08.08.2014	1.2.3.38.38.38.367
39455	SRR1966692	SAMN03476770	Human	England	30.07.2014	1.2.3.38.38.38.367
39454	SRR1969334	SAMN03479411	Human	England	28.07.2014	1.2.3.38.38.38.367
39453	SRR1966194	SAMN03476278	Human	England	29.07.2014	1.2.3.38.38.38.367
39452	SRR1969276	SAMN03479353	Human	England	28.07.2014	1.2.3.38.38.38.367
39450	SRR1967179	SAMN03477269	Human	England	23.07.2014	1.2.3.38.38.38.367
39449	SRR1966447	SAMN03476530	Human	England	21.07.2014	1.2.3.38.38.38.367
38766	SRR1965590	SAMN03475705	Human	England	05.08.2014	1.2.3.38.38.38.367
38430	SRR1969891	SAMN03479968	Human	Jersey	01.08.2014	1.2.3.38.38.38.367
38426	SRR1965596	SAMN03475709	Human	England	28.07.2014	1.2.3.38.38.38.367
38371	SRR1967766	SAMN03477856	Human	England	28.07.2014	1.2.3.38.38.38.367
38369	SRR1968929	SAMN03479027	Human	England	30.07.2014	1.2.3.38.38.38.367
38368	SRR3120716	SAMN04440811	Human	England	31.07.2014	1.2.3.38.38.38.367
38361	SRR1965359	SAMN03475482	Human	England	28.07.2014	1.2.3.38.38.38.367
38325	SRR1968832	SAMN03478930	Human	England	30.07.2014	1.2.3.38.38.38.367
38318	SRR1966486	SAMN03476569	Human	England	28.07.2014	1.2.3.38.38.38.367
38317	SRR1967378	SAMN03477468	Human	England	28.07.2014	1.2.3.38.38.38.367
38316	SRR1966755	SAMN03476833	Human	England	28.07.2014	1.2.3.38.38.38.367
38315	SRR1969298	SAMN03479375	Human	England	28.07.2014	1.2.3.38.38.38.367
38314	SRR1965369	SAMN03475492	Human	England	29.07.2014	1.2.3.38.38.38.367
38313	SRR1966969	SAMN03477047	Human	England	28.07.2014	1.2.3.38.38.38.367
38311	SRR1965464	SAMN03475585	Human	England	29.07.2014	1.2.3.38.38.38.367
37834	SRR1969400	SAMN03479477	Human	England	31.07.2014	1.2.3.38.38.38.367
37833	SRR1966923	SAMN03477001	Human	England	30.07.2014	1.2.3.38.38.38.367
37831	SRR1966129	SAMN03476214	Human	England	30.07.2014	1.2.3.38.38.38.367

37830	SRR1970182	SAMN03480258	Human	England	30.07.2014	1.2.3.38.38.38.367
37829	SRR1969802	SAMN03479878	Human	England	28.07.2014	1.2.3.38.38.38.367
37828	SRR1965700	SAMN03475808	Human	England	28.07.2014	1.2.3.38.38.38.367
37827	SRR1968120	SAMN03478211	Human	England	24.07.2014	1.2.3.38.38.38.367
37826	SRR1969838	SAMN03479915	Human	England	28.07.2014	1.2.3.38.38.38.367
37824	SRR1970294	SAMN03480370	Human	England	27.07.2014	1.2.3.38.38.38.367
37817	SRR1968529	SAMN03478622	Human	England	26.07.2014	1.2.3.38.38.38.367
37815	SRR1969805	SAMN03479881	Human	England	30.07.2014	1.2.3.38.38.38.367
37814	SRR1967038	SAMN03477116	Human	England	30.07.2014	1.2.3.38.38.38.367
37813	SRR1968627	SAMN03478719	Human	England	31.07.2014	1.2.3.38.38.38.367
37812	SRR1966507	SAMN03476590	Human	England	31.07.2014	1.2.3.38.38.38.367
37811	SRR1968796	SAMN03478894	Human	England	28.07.2014	1.2.3.38.38.38.367
37810	SRR1968043	SAMN03478134	Human	England	29.07.2014	1.2.3.38.38.38.367
37809	SRR1967479	SAMN03477569	Human	England	29.07.2014	1.2.3.38.38.38.367
37808	SRR1965800	SAMN03475898	Human	England	31.07.2014	1.2.3.38.38.38.367
37800	SRR1968900	SAMN03478998	Human	England	29.07.2014	1.2.3.38.38.38.367
37799	SRR1969732	SAMN03479808	Human	England	01.08.2014	1.2.3.38.38.38.367
37798	SRR1969301	SAMN03479378	Human	England	28.07.2014	1.2.3.38.38.38.367
37778	SRR1965530	SAMN03475652	Human	England	01.08.2014	1.2.3.38.38.38.367
36530	SRR1969546	SAMN03479623	Human	England	28.07.2014	1.2.3.38.38.38.367
36526	SRR1967771	SAMN03477861	Human	England	28.07.2014	1.2.3.38.38.38.367
36524	SRR1967089	SAMN03477167	Human	England	22.07.2014	1.2.3.38.38.38.367
36521	SRR1966349	SAMN03476432	Human	England	24.07.2014	1.2.3.38.38.38.367
36518	SRR1969134	SAMN03479211	Human	England	23.07.2014	1.2.3.38.38.38.367
36516	SRR1969758	SAMN03479834	Human	England	21.07.2014	1.2.3.38.38.38.367
36498	SRR1966605	SAMN03476686	Human	England	28.07.2014	1.2.3.38.38.38.367
36477	SRR1968778	SAMN03478876	Other	England	21.07.2014	1.2.3.38.38.38.367
36474	SRR1969839	SAMN03479916	Other	England	21.07.2014	1.2.3.38.38.38.367

36464	SRR1967609	SAMN03477699	Human	England	25.07.2014	1.2.3.38.38.38.367
36395	SRR1969024	SAMN03479120	Human	England	03.08.2014	1.2.3.38.38.38.367
36394	SRR1967993	SAMN03478084	Human	England	04.08.2014	1.2.3.38.38.38.367
34199	SRR1965044	SAMN03473942	Human	England	24.07.2014	1.2.3.38.38.38.367
34197	SRR1967286	SAMN03477376	Human	England	24.07.2014	1.2.3.38.38.38.367
34196	SRR1967185	SAMN03477275	Human	England	25.07.2014	1.2.3.38.38.38.367
34190	SRR3049621	SAMN04363906	Human	England	24.07.2014	1.2.3.38.38.38.367
34189	SRR1966851	SAMN03476929	Human	England	25.07.2014	1.2.3.38.38.38.367
34188	SRR1968592	SAMN03478684	Human	England	25.07.2014	1.2.3.38.38.38.367
34187	SRR1966252	SAMN03476335	Human	England	25.07.2014	1.2.3.38.38.38.367
34186	SRR1967080	SAMN03477158	Human	England	25.07.2014	1.2.3.38.38.38.367
34182	SRR1970179	SAMN03480255	Human	England	24.07.2014	1.2.3.38.38.38.367
32525	SRR1967216	SAMN03477306	Human	England	18.07.2014	1.2.3.38.38.38.367
32472	SRR1967085	SAMN03477163	Human	England	18.07.2014	1.2.3.38.38.38.367
32471	SRR1969002	SAMN03479099	Human	England	20.07.2014	1.2.3.38.38.38.367
32470	SRR1966685	SAMN03476763	Human	England	17.07.2014	1.2.3.38.38.38.367
32463	SRR1967462	SAMN03477552	Human	England	17.07.2014	1.2.3.38.38.38.367
32462	SRR1966087	SAMN03476173	Human	England	15.07.2014	1.2.3.38.38.38.367
32460	SRR1969597	SAMN03479673	Human	England	16.07.2014	1.2.3.38.38.38.367
32459	SRR1966830	SAMN03476908	Human	England	17.07.2014	1.2.3.38.38.38.367
31981	SRR1970163	SAMN03480239	Human	England	25.07.2014	1.2.3.38.38.38.367
31980	SRR1966240	SAMN03476323	Human	England	25.07.2014	1.2.3.38.38.38.367
31972	SRR1968564	SAMN03478657	Human	England	25.07.2014	1.2.3.38.38.38.367
31968	SRR1967858	SAMN03477949	Human	England	26.07.2014	1.2.3.38.38.38.367
31967	SRR1966151	SAMN03476236	Human	England	26.07.2014	1.2.3.38.38.38.367
31966	SRR1968746	SAMN03478844	Human	England	25.07.2014	1.2.3.38.38.38.367
31965	SRR1968329	SAMN03478420	Human	England	25.07.2014	1.2.3.38.38.38.367
31929	SRR1965679	SAMN03475789	Human	England	21.07.2014	1.2.3.38.38.38.367

31928	SRR1966387	SAMN03476470	Human	England	21.07.2014	1.2.3.38.38.38.367
31927	SRR1970220	SAMN03480296	Human	England	22.07.2014	1.2.3.38.38.38.367
31923	SRR1967548	SAMN03477638	Human	England	23.07.2014	1.2.3.38.38.38.367
31922	SRR1966554	SAMN03476636	Human	England	21.07.2014	1.2.3.38.38.38.367
29972	SRR3049720	SAMN04363988	Human	England	21.07.2014	1.2.3.38.38.38.367
29971	SRR3049565	SAMN04363857	Human	England	21.07.2014	1.2.3.38.38.38.367
29970	SRR3048933	SAMN04363178	Human	England	21.07.2014	1.2.3.38.38.38.367
68909	SRR1966478	SAMN03476561	Human	England	24.06.2014	1.2.3.38.38.38.38
53036	SRR3049067	SAMN04363302	Human	England	23.09.2014	1.2.3.38.38.38.38
31623	SRR1968972	SAMN03479070	Human	England	09.07.2014	1.2.3.38.38.38.38
31613	SRR1969753	SAMN03479829	Human	England	14.07.2014	1.2.3.38.38.38.38
27797	SRR1969004	SAMN03479101	Human	England	27.06.2014	1.2.3.38.38.38.38
27067	SRR1966984	SAMN03477062	Human	England	07.07.2014	1.2.3.38.38.38.38
25341	SRR1965266	SAMN03475391	Human	England	25.06.2014	1.2.3.38.38.38.38
23493	SRR1969003	SAMN03479100	Human	England	19.06.2014	1.2.3.38.38.38.38
23472	SRR1967160	SAMN03477250	Human	England	13.06.2014	1.2.3.38.38.38.38
23462	SRR1968861	SAMN03478959	Human	England	10.06.2014	1.2.3.38.38.38.38
21846	SRR3049191	SAMN04363433	Human	England	09.06.2014	1.2.3.38.38.38.38
21845	SRR3048579	SAMN04362947	Human	England	10.06.2014	1.2.3.38.38.38.38
21844	SRR3049723	SAMN04363991	Human	England	10.06.2014	1.2.3.38.38.38.38
21843	SRR3049661	SAMN04363938	Human	England	01.06.2014	1.2.3.38.38.38.38
21815	SRR3049937	SAMN04364182	Human	England	09.06.2014	1.2.3.38.38.38.38
21810	SRR3048785	SAMN04363036	Human	England	06.06.2014	1.2.3.38.38.38.38
21809	SRR3401403	SAMN04868780	Enviromental	England	08.06.2014	1.2.3.38.38.38.38
21807	SRR3323014	SAMN04601098	Enviromental	England	08.06.2014	1.2.3.38.38.38.38
21786	SRR1966858	SAMN03476936	Human	England	03.06.2014	1.2.3.38.38.38.38
21785	SRR1965313	SAMN03475437	Human	England	03.06.2014	1.2.3.38.38.38.38
21783	SRR1969436	SAMN03479513	Human	England	05.06.2014	1.2.3.38.38.38.38

21782	SRR1968806	SAMN03478904	Human	England	02.06.2014	1.2.3.38.38.38.38
21779	SRR1969526	SAMN03479603	Human	England	12.06.2014	1.2.3.38.38.38.38
21778	SRR1966448	SAMN03476531	Human	England	12.06.2014	1.2.3.38.38.38.38
21777	SRR1965549	SAMN03475671	Human	England	12.06.2014	1.2.3.38.38.38.38
21776	SRR1967099	SAMN03477177	Human	England	03.06.2014	1.2.3.38.38.38.38
21770	SRR1967228	SAMN03477318	Human	England	06.06.2014	1.2.3.38.38.38.38
21117	SRR3049094	SAMN04363330	Human	England	04.06.2014	1.2.3.38.38.38.38
21115	SRR3049939	SAMN04364183	Human	England	04.06.2014	1.2.3.38.38.38.38
21114	SRR3049844	SAMN04364099	Human	England	04.06.2014	1.2.3.38.38.38.38
20927	SRR3049543	SAMN04363836	Human	England	08.06.2014	1.2.3.38.38.38.38
20925	SRR3049443	SAMN04363739	Human	England	06.06.2014	1.2.3.38.38.38.38
20909	SRR3049104	SAMN04363341	Human	England	06.04.2014	1.2.3.38.38.38.38
18557	SRR3048580	SAMN04362948	Human	England	06.06.2014	1.2.3.38.38.38.38
18556	SRR3049743	SAMN04364003	Human	England	06.06.2014	1.2.3.38.38.38.38
18555	SRR3049265	SAMN04363554	Human	England	06.06.2014	1.2.3.38.38.38.38
18554	SRR3049366	SAMN04363660	Human	England	06.06.2014	1.2.3.38.38.38.38
21767	SRR1967796	SAMN03477886	Human	England	04.06.2014	1.2.3.38.38.38.38
46586	SRR1969069	SAMN03479163	Human	England	14.07.2014	1.2.3.38.38.38.393
46585	SRR1966383	SAMN03476466	Human	England	18.07.2014	1.2.3.38.38.38.393
46584	SRR1970226	SAMN03480302	Human	England	17.07.2014	1.2.3.38.38.38.393
46583	SRR1969372	SAMN03479449	Human	England	14.07.2014	1.2.3.38.38.38.393
46582	SRR1967046	SAMN03477124	Human	England	16.07.2014	1.2.3.38.38.38.393
39446	SRR1970141	SAMN03480217	Human	England	12.07.2014	1.2.3.38.38.38.393
38345	SRR1970302	SAMN03480378	Human	England	28.07.2014	1.2.3.38.38.38.393
38344	SRR1968654	SAMN03478752	Human	England	28.07.2014	1.2.3.38.38.38.393
38319	SRR1966971	SAMN03477049	Human	England	28.07.2014	1.2.3.38.38.38.393
37823	SRR1965424	SAMN03475546	Human	England	30.07.2014	1.2.3.38.38.38.393
37822	SRR1966491	SAMN03476574	Human	England	30.07.2014	1.2.3.38.38.38.393

37007	SRR1969845	SAMN03479922	Human	England	01.08.2014	1.2.3.38.38.38.393
36525	SRR1966937	SAMN03477015	Human	England	24.07.2014	1.2.3.38.38.38.393
36501	SRR1967002	SAMN03477080	Human	England	17.07.2014	1.2.3.38.38.38.393
34203	SRR1968423	SAMN03478515	Food	England	21.07.2014	1.2.3.38.38.38.393
34202	SRR1967187	SAMN03477277	Enviromental	England	21.07.2014	1.2.3.38.38.38.393
34194	SRR1966724	SAMN03476802	Human	England	24.07.2014	1.2.3.38.38.38.393
34185	SRR1967174	SAMN03477264	Human	England	22.07.2014	1.2.3.38.38.38.393
34184	SRR1967383	SAMN03477473	Human	England	22.07.2014	1.2.3.38.38.38.393
34181	SRR1969076	SAMN03479169	Human	England	22.07.2014	1.2.3.38.38.38.393
34179	SRR1969206	SAMN03479283	Human	England	21.07.2014	1.2.3.38.38.38.393
34178	SRR1966339	SAMN03476422	Human	England	17.07.2014	1.2.3.38.38.38.393
34177	SRR1968906	SAMN03479004	Human	England	22.07.2014	1.2.3.38.38.38.393
34176	SRR1965958	SAMN03476049	Human	England	21.07.2014	1.2.3.38.38.38.393
32550	SRR1969229	SAMN03479306	Human	England	18.07.2014	1.2.3.38.38.38.393
32523	SRR1965375	SAMN03475498	Human	England	15.07.2014	1.2.3.38.38.38.393
32478	SRR1969172	SAMN03479249	Other	England	17.07.2014	1.2.3.38.38.38.393
32477	SRR1966289	SAMN03476372	Food	England	17.07.2014	1.2.3.38.38.38.393
32476	SRR1965122	SAMN03474017	Food	England	17.07.2014	1.2.3.38.38.38.393
32466	SRR1967989	SAMN03478080	Human	England	18.07.2014	1.2.3.38.38.38.393
32375	SRR1967533	SAMN03477623	Human	England	18.07.2014	1.2.3.38.38.38.393
31976	SRR1965987	SAMN03476078	Human	England	23.07.2014	1.2.3.38.38.38.393
31974	SRR1967787	SAMN03477877	Human	England	25.07.2014	1.2.3.38.38.38.393
31973	SRR1966105	SAMN03476190	Human	England	22.07.2014	1.2.3.38.38.38.393
31652	SRR1969601	SAMN03479677	Human	England	09.07.2014	1.2.3.38.38.38.393
29391	SRR1970288	SAMN03480364	Human	England	14.07.2014	1.2.3.38.38.38.393
29281	SRR3049409	SAMN04363704	Human	England	11.07.2014	1.2.3.38.38.38.393
29280	SRR3049070	SAMN04363305	Human	England	11.07.2014	1.2.3.38.38.38.393
31612	SRR1969738	SAMN03479814	Human	England	14.07.2014	1.2.3.38.38.38.394

31611	SRR1967131	SAMN03477221	Human	England	14.07.2014	1.2.3.38.38.38.394
40793	SRR1965094	SAMN03473990	Human	England	12.08.2014	1.2.3.38.38.38.395
91893	SRR1968044	SAMN03478135	Human	England	01.08.2014	1.2.3.38.38.38.413
65449	SRR1965735	SAMN03475840	Enviromental	England	01.08.2014	1.2.3.38.38.38.413
57113	SRR1966345	SAMN03476428	Human	England	05.08.2014	1.2.3.38.38.38.413
57112	SRR1965784	SAMN03475884	Food	England	01.08.2014	1.2.3.38.38.38.413
57110	SRR1969068	SAMN03479162	Enviromental	England	01.08.2014	1.2.3.38.38.38.413
57103	SRR1967394	SAMN03477484	Human	England	31.07.2014	1.2.3.38.38.38.413
56935	SRR3048716	SAMN04362990	Human	England	01.08.2014	1.2.3.38.38.38.413
53063	SRR1969068	SAMN03479162	Enviromental	England	01.08.2014	1.2.3.38.38.38.413
52972	SRR1967076	SAMN03477154	Human	England	26.09.2014	1.2.3.38.38.38.413
50361	SRR1965353	SAMN03475476	Human	England	04.09.2014	1.2.3.38.38.38.413
50158	SRR1958208	SAMN03466101	Human	England	20.09.2014	1.2.3.38.38.38.413
43909	SRR3048844	SAMN04363095	Human	England	21.08.2014	1.2.3.38.38.38.413
41995	SRR1969283	SAMN03479360	Human	England	20.08.2014	1.2.3.38.38.38.413
40235	SRR1968516	SAMN03478609	Human	England	12.08.2014	1.2.3.38.38.38.413
40224	SRR1968520	SAMN03478613	Human	England	13.08.2014	1.2.3.38.38.38.413
39514	SRR1969190	SAMN03479267	Human	England	14.08.2014	1.2.3.38.38.38.413
38779	SRR1969416	SAMN03479493	Human	England	07.08.2014	1.2.3.38.38.38.413
38773	SRR1970014	SAMN03480092	Human	England	07.08.2014	1.2.3.38.38.38.413
38772	SRR1966715	SAMN03476793	Human	England	06.08.2014	1.2.3.38.38.38.413
38422	SRR1968589	SAMN03478681	Human	England	25.07.2014	1.2.3.38.38.38.413
38421	SRR1965623	SAMN03475736	Human	England	29.07.2014	1.2.3.38.38.38.413
38420	SRR1965410	SAMN03475532	Human	England	29.07.2014	1.2.3.38.38.38.413
38419	SRR1967028	SAMN03477106	Human	England	29.07.2014	1.2.3.38.38.38.413
38418	SRR1965458	SAMN03475580	Human	England	30.07.2014	1.2.3.38.38.38.413
38417	SRR1968051	SAMN03478142	Human	England	28.07.2014	1.2.3.38.38.38.413
38309	SRR1965496	SAMN03475617	Human	England	28.07.2014	1.2.3.38.38.38.413

37819	SRR1965533	SAMN03475655	Human	England	29.07.2014	1.2.3.38.38.38.413
37047	SRR3417495	SAMN04884707	Human	Austria	2014	1.2.3.38.38.38.413
29308	SRR3049775	SAMN04364027	Human	England	15.07.2014	1.2.3.38.38.38.413
23057	SRR1968791	SAMN03478889	Human	England	07.06.2014	1.2.3.38.38.38.413
36528	SRR1969692	SAMN03479768	Human	England	28.07.2014	1.2.3.38.38.38.436
74309	SRR1963071	SAMN03469619	Human	England	27.11.2014	1.2.3.38.38.38.458
43431	SRR1965484	SAMN03475605	Human	England	29.08.2014	1.2.3.38.38.38.458
62574	SRR1957852	SAMN03465754	Human	England	01.09.2014	1.2.3.38.38.38.461
46155	SRR3049322	SAMN04363617	Human	England	27.08.2014	1.2.3.38.38.38.461
45262	SRR1957814	SAMN03465710	Human	England	01.09.2014	1.2.3.38.38.38.461
43427	SRR1969209	SAMN03479286	Human	England	28.08.2014	1.2.3.38.38.38.461
36506	SRR1965669	SAMN03475780	Human	England	24.07.2014	1.2.3.38.38.38.461
27771	SRR1969349	SAMN03479426	Human	England	26.06.2014	1.2.3.38.38.38.472
26644	SRR1966029	SAMN03476116	Human	England	30.06.2014	1.2.3.38.38.38.472
26641	SRR1969340	SAMN03479417	Human	England	26.06.2014	1.2.3.38.38.38.472
25320	SRR1968299	SAMN03478390	Human	England	24.06.2014	1.2.3.38.38.38.472
25225	SRR1969895	SAMN03479972	Human	England	28.07.2014	1.2.3.38.38.38.472
24987	SRR1967540	SAMN03477630	Human	England	28.06.2014	1.2.3.38.38.38.472
24986	SRR1967307	SAMN03477397	Human	England	28.06.2014	1.2.3.38.38.38.472
24039	SRR1969015	SAMN03479112	Human	England	23.06.2014	1.2.3.38.38.38.472
24038	SRR1967780	SAMN03477870	Human	England	23.06.2014	1.2.3.38.38.38.472
40794	SRR1966307	SAMN03476390	Human	England	12.08.2014	1.2.3.38.38.38.478
38410	SRR1970082	SAMN03480159	Human	England	01.08.2014	1.2.3.38.38.38.483
69743	SRR1960032	SAMN03468764	Human	England	13.11.2014	1.2.3.38.38.38.484
36392	SRR1966502	SAMN03476585	Human	England	04.08.2014	1.2.3.38.38.38.488
43927	SRR3120707	SAMN04440804	Human	England	17.08.2014	1.2.3.38.38.38.515
54008	SRR3417503	SAMN04884715	Food	Germany	2014	1.2.3.38.38.38.533
31656	SRR1968675	SAMN03478773	Human	England	10.07.2014	1.2.3.38.38.38.533

34175	SRR1968899	SAMN03478997	Human	England	22.07.2014	1.2.3.38.38.38.537
68636	SRR1958280	SAMN03466173	Human	England	13.10.2014	1.2.3.38.38.38.544
66845	SRR1958280	SAMN03466173	Human	England	13.10.2014	1.2.3.38.38.38.544
38411	SRR1967261	SAMN03477351	Human	England	30.07.2014	1.2.3.38.38.38.544
36504	SRR1966077	SAMN03476163	Human	England	23.07.2014	1.2.3.38.38.38.544
36385	SRR1967625	SAMN03477715	Human	England	04.08.2014	1.2.3.38.38.38.544
32464	SRR1967729	SAMN03477819	Human	England	17.07.2014	1.2.3.38.38.38.544
201405760	SRR3410220	SAMN04883178	Human	France	2014	1.2.3.38.38.38.550
62581	SRR1957877	SAMN03465779	Human	England	27.08.2014	1.2.3.38.38.38.550
62554	SRR1958460	SAMN03466353	Human	England	24.08.2014	1.2.3.38.38.38.550
48206	SRR1958131	SAMN03466031	Human	England	04.09.2014	1.2.3.38.38.38.550
48161	SRR3049170	SAMN04363408	Human	England	24.08.2014	1.2.3.38.38.38.550
46156	SRR1958546	SAMN03466439	Human	England	19.08.2014	1.2.3.38.38.38.550
46125	SRR3048951	SAMN04363194	Human	England	23.08.2014	1.2.3.38.38.38.550
46118	SRR1957986	SAMN03465888	Human	England	20.08.2014	1.2.3.38.38.38.550
46085	SRR1958028	SAMN03465929	Human	England	19.08.2014	1.2.3.38.38.38.550
46009	SRR1965753	SAMN03475855	Human	England	09.08.2014	1.2.3.38.38.38.550
45269	SRR3049365	SAMN04363659	Human	England	27.08.2014	1.2.3.38.38.38.550
43960	SRR3049722	SAMN04363990	Human	England	18.08.2014	1.2.3.38.38.38.550
43958	SRR3049513	SAMN04363806	Human	England	18.08.2014	1.2.3.38.38.38.550
43937	SRR3049811	SAMN04364054	Human	England	21.08.2014	1.2.3.38.38.38.550
41991	SRR1969928	SAMN03480005	Human	England	19.08.2014	1.2.3.38.38.38.550
40327	SRR1965444	SAMN03475566	Human	England	18.08.2014	1.2.3.38.38.38.550
40324	SRR1967321	SAMN03477411	Human	England	18.08.2014	1.2.3.38.38.38.550
39517	SRR3049549	SAMN04363842	Human	England	12.08.2014	1.2.3.38.38.38.550
39515	SRR1967284	SAMN03477374	Human	England	11.08.2014	1.2.3.38.38.38.550
38428	SRR1969578	SAMN03479655	Human	England	29.07.2014	1.2.3.38.38.38.550
31952	SRR1965867	SAMN03475961	Human	England	24.07.2014	1.2.3.38.38.38.550

25266	SRR1968651	SAMN03478749	Human	England	01.07.2014	1.2.3.38.38.38.550
25260	SRR1966102	SAMN03476188	Human	England	07.07.2014	1.2.3.38.38.38.550
25004	SRR3049783	SAMN04364033	Human	England	03.07.2014	1.2.3.38.38.38.550
20929	SRR3049502	SAMN04363795	Human	England	03.06.2014	1.2.3.38.38.38.550
39447	SRR1967990	SAMN03478081	Human	England	18.07.2014	1.2.3.38.38.38.570
37759	SRR1965339	SAMN03475462	Human	England	28.07.2014	1.2.3.38.38.38.570
61859	SRR1967169	SAMN03477259	Human	England	10.10.2014	1.2.3.38.38.38.571
37036	SRR1968931	SAMN03479029	Human	France	2014	1.2.3.38.38.38.600
24040	SRR1967283	SAMN03477373	Human	England	20.06.2014	1.2.3.38.38.38.604
50356	SRR1965213	SAMN03475345	Human	England	05.09.2014	1.2.3.38.38.38.624
34180	SRR1965068	SAMN03473965	Human	England	17.07.2014	1.2.3.38.38.38.624
31969	SRR1965895	SAMN03475988	Human	England	25.07.2014	1.2.3.38.38.38.624
69699	SRR1963272	SAMN03469731	Human	England	12.11.2014	1.2.3.38.38.38.653
65390	SRR1967121	SAMN03477211	Human	England	12.09.2014	1.2.3.38.38.38.653
65125	SRR1969192	SAMN03479269	Human	England	04.11.2014	1.2.3.38.38.38.653
62610	SRR1958272	SAMN03466165	Human	England	30.10.2014	1.2.3.38.38.38.653
57130	SRR1967597	SAMN03477687	Human	England	30.09.2014	1.2.3.38.38.38.653
50360	SRR1967801	SAMN03477891	Human	England	03.09.2014	1.2.3.38.38.38.653
37006	SRR1967999	SAMN03478090	Human	England	03.08.2014	1.2.3.38.38.38.653
65434	SRR1968603	SAMN03478695	Human	England	30.09.2014	1.2.3.38.38.38.655
60074	SRR1958676	SAMN03466569	Human	England	09.10.2014	1.2.3.38.38.38.655
58510	SRR1958011	SAMN03465913	Human	England	02.10.2014	1.2.3.38.38.38.655
57641	SRR1958219	SAMN03466111	Human	England	26.09.2014	1.2.3.38.38.38.655
52954	SRR1965327	SAMN03475451	Human	England	25.09.2014	1.2.3.38.38.38.655
31917	SRR1968213	SAMN03478304	Human	England	18.07.2014	1.2.3.38.38.38.668
37035	SRR1969713	SAMN03479789	Human	France	2014	1.2.3.38.38.38.673
14-05227	SRR3410211	SAMN04883167	Enviromental	Germany	2014	1.2.3.38.38.38.685
14-05224	SRR3410208	SAMN04883163	Enviromental	Germany	2014	1.2.3.38.38.38.686

14-05225	SRR3410209	SAMN04883164	Enviromental	Germany	2014	1.2.3.38.38.38.687
32467	SRR1969951	SAMN03480029	Human	England	19.07.2014	1.2.3.38.38.38.703
65379	SRR1965774	SAMN03475875	Human	England	29.09.2014	1.2.3.38.38.38.728
21781	SRR1966482	SAMN03476565	Human	England	02.06.2014	1.2.3.38.38.38.729
36476	SRR1967478	SAMN03477568	Other	England	21.07.2014	1.2.3.38.38.38.746
36475	SRR1968659	SAMN03478757	Other	England	21.07.2014	1.2.3.38.38.38.746
36473	SRR1965618	SAMN03475731	Other	England	21.07.2014	1.2.3.38.38.38.746
201405861	SRR3410221	SAMN04883179	Human	France	2014	1.2.3.38.38.38.748
40795	SRR1968912	SAMN03479010	Human	England	15.08.2014	1.2.3.38.38.38.766
40274	SRR1968614	SAMN03478706	Human	England	11.08.2014	1.2.3.38.38.38.766
50109	SRR1958033	SAMN03465934	Human	England	16.09.2014	1.2.3.38.38.38.787
65373	SRR1968069	SAMN03478160	Human	England	02.10.2014	1.2.3.38.38.38.790
50104	SRR3417500	SAMN04884712	Human	Austria	2014	1.2.3.38.38.38.790
45605	SRR3417496	SAMN04884708	Human	Luxembourg	2014	1.2.3.38.38.38.790
31977	SRR1965563	SAMN03475686	Human	England	22.04.2014	1.2.3.38.38.38.790
21871	SRR3049904	SAMN04364150	Human	England	12.06.2014	1.2.3.38.38.38.807
37825	SRR1968556	SAMN03478649	Human	England	28.07.2014	1.2.3.38.38.38.808
36364	SRR1968553	SAMN03478646	Human	England	30.07.2014	1.2.3.38.38.38.910
23500	SRR1966002	SAMN03476093	Human	England	17.06.2014	1.2.3.38.38.38.949
21120	SRR3049851	SAMN04364105	Human	England	02.06.2014	1.2.3.38.38.38.949
14128	SRR1966935	SAMN03477013	Human	England	22.05.2014	1.2.3.38.38.38.949
14099	SRR1967422	SAMN03477512	Human	England	21.05.2014	1.2.3.38.38.38.949
12110	SRR1969291	SAMN03479368	Human	England	08.05.2014	1.2.3.38.38.38.949
23507	SRR1969932	SAMN03480009	Human	England	19.06.2014	1.2.3.38.38.38.962
13389	SRR1968417	SAMN03478509	Human	England	12.05.2014	1.2.3.38.38.38.962
73221	SRR1966911	SAMN03476989	Human	England	08.12.2014	1.2.3.38.38.38.970
73639	SRR1960236	SAMN03469037	Human	England	10.12.2014	1.21.152.270.270.270.270
49744	SRR1957853	SAMN03465755	Human	England	23.09.2014	1.21.92.155.155.155.155

66859	SRR1958121	SAMN03466021	Human	England	24.10.2014	1.24.129.235.235.235.235
70657	SRR1959226	SAMN03468429	Human	England	27.11.2014	1.25.140.251.251.251.251
71396	SRR1959443	SAMN03468624	Human	England	06.11.2012	1.26.144.258.258.258.258
1514	SRR3049796	SAMN04364040	Human	England	25.03.2014	1.27.153.271.271.271.271
170334	SRR3285470	SAMN04575166	Human	England	17.09.2015	1.27.225.1150.1907.2277.3665
5355	SRR1966351	SAMN03476434	Human	England	02.04.2014	1.3.154.272.272.272.272
104253	SRR3286602	SAMN04576284	Human	England	20.04.2015	1.3.224.1021.1652.1906.2677
120578	SRR3585357	SAMN05173211	Human	England	02.06.2015	1.3.235.1046.1710.1985.2905
146116	SRR3285416	SAMN04575061	Human	England	12.08.2015	1.3.249.1082.1788.2113.3276
164715	SRR3585351	SAMN05173206	Human	England	21.09.2015	1.3.263.1128.1873.2229.3531
9310	SRR1968303	SAMN03478394	Human	England	23.04.2014	1.3.6.6.6.6.6
154869	SRR3585356	SAMN05173210	Human	England	01.09.2015	1.47.260.1114.1845.2190.3444
62551	SRR1957933	SAMN03465835	Human	England	03.09.2014	1.5.112.204.204.204.204
9263	SRR1968125	SAMN03478216	Human	England	24.04.2014	1.5.159.280.280.280.280
H122280366	SRR1645898	SAMN03169186	Human	England	29.05.2012	1.5.166.294.294.294.294
H122540669-1	SRR1645877	SAMN03169165	Human	England	16.06.2012	1.5.170.299.299.299.299
83706	SRR1969162	SAMN03479239	Human	England	04.12.2014	1.5.192.373.396.403.786
80341	SRR1960928	SAMN03469143	Human	England	13.01.2015	1.5.201.507.639.686.839
80363	SRR1960665	SAMN03469130	Human	England	14.01.2015	1.5.205.604.822.905.1161
94103	SRR1969779	SAMN03479855	Human	England	05.03.2015	1.5.209.638.889.994.1289
178732	SRR3585363	SAMN05173221	Human	England	13.10.2015	1.5.276.1175.1987.2378.3879
183605	SRR3585364	SAMN05173222	Human	England	02.11.2015	1.5.279.1188.2016.2426.4013
40280	SRR1968399	SAMN03478490	Human	England	19.08.2014	1.5.69.111.111.111.111
40366	SRR1968732	SAMN03478830	Human	England	28.08.2014	1.5.72.115.115.115.115
44692	SRR1957809	SAMN03465705	Human	England	08.09.2014	1.5.79.129.129.129.129
45606	SRR1965360	SAMN03475483	Human	England	09.09.2014	1.5.83.136.136.136.136
12185	SRR1967057	SAMN03477135	Human	England	13.05.2014	1.5.9.11.11.11.11
H123940649	SRR1635114	SAMN03152416	Human	England	21.09.2012	1.6.11.13.333.333.333

14875	SRR1966084	SAMN03476170	Human	England	20.05.2014	1.7.14.16.16.16.16
71409	SRR1960073	SAMN03468915	Human	England	06.11.2012	1.7.146.262.262.262.262
44755	SRR1957732	SAMN03465627	Human	England	10.09.2014	10.20.81.133.133.133.133
H121440322	SRR1645334	SAMN03168724	Human	England	26.03.2012	13.29.164.288.288.288.288
193289	SRR3585377	SAMN05173228	Food	England	02.12.2015	13.52.281.1202.2042.2470.4123
99074	SRR3049054	SAMN04363288	Human	England	27.03.2015	15.32.196.474.586.621.745
95025	SRR1970156	SAMN03480232	Human	England	11.03.2015	17.38.223.1020.1637.1891.2642
190569	SRR3585365	SAMN05173223	Human	England	24.11.2015	20.53.283.1206.2050.2481.4147
37836	SRR1968725	SAMN03478823	Human	England	05.08.2014	3.13.56.93.93.93.93
40348	SRR1970029	SAMN03480107	Human	England	28.08.2014	3.17.71.114.114.114.114
55582	SRR1966056	SAMN03476142	Human	England	19.09.2014	3.4.104.177.177.177.177
69142	SRR1963148	SAMN03469677	Human	England	13.11.2014	3.4.137.245.245.245.245
70664	SRR1963220	SAMN03469683	Human	England	27.11.2014	3.4.141.253.253.253.253
131456	SRR3585361	SAMN05173220	Human	England	08.07.2015	3.4.241.1063.1740.2042.3081
H123480474	SRR1645784	SAMN03169050	Human	England	21.08.2012	3.4.49.318.318.318.318
62560	SRR1957878	SAMN03465780	Human	England	05.09.2014	3.4.8.205.205.205.205
34201	SRR1968131	SAMN03478222	Human	England	29.07.2014	3.4.8.81.81.81.81
83318	SRR1965963	SAMN03476054	Human	England	21.01.2015	4.31.193.423.488.506.568
160846	SRR3585359	SAMN05173214	Human	England	14.09.2015	4.31.264.1129.1875.2231.3537
137147	SRR3585354	SAMN05173208	Human	England	23.07.2015	4.44.246.1071.1757.2064.3146
153189	SRR3585358	SAMN05173212	Human	England	27.08.2015	4.45.257.1106.1833.2173.3394
168519	SRR3585375	SAMN05173226	Human	England	25.09.2015	4.50.271.1154.1912.2284.3677
14895	SRR1967335	SAMN03477425	Human	England	21.05.2014	4.8.15.17.17.17.17
18577	SRR3049240	SAMN04363526	Human	England	06.06.2014	4.9.21.24.24.24.24
53007	SRR1965781	SAMN03475881	Human	England	01.10.2014	8.14.59.168.168.168.168
63923	SRR1970142	SAMN03480218	Human	England	06.11.2014	8.23.121.220.220.220.220
46132	SRR1957863	SAMN03465765	Human	England	29.08.2014	9.16.68.108.144.144.144

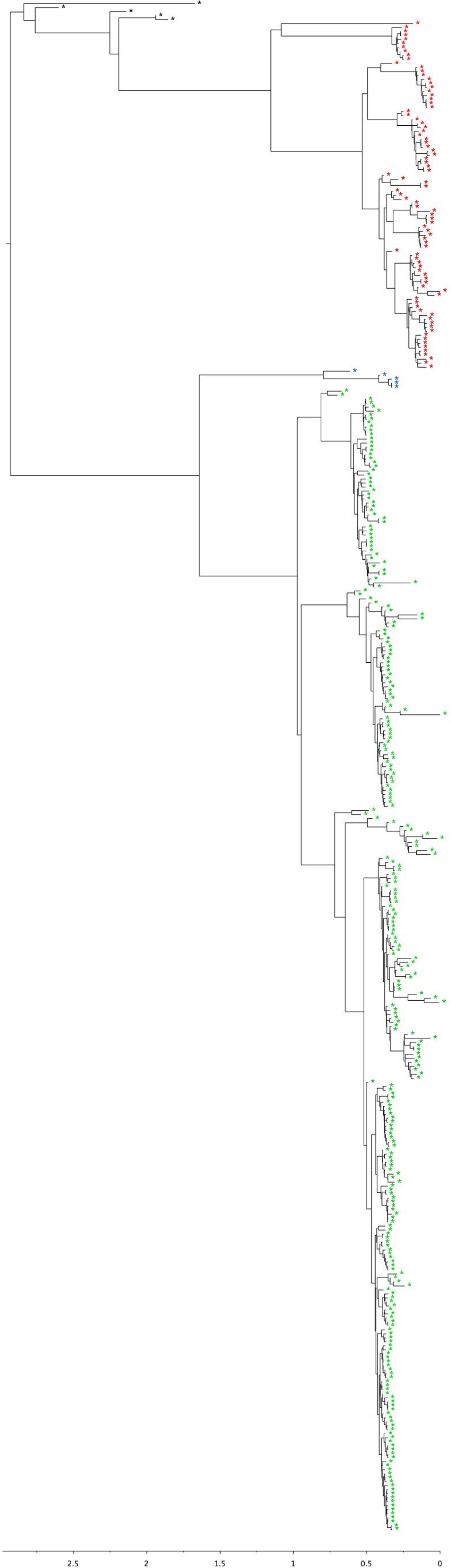
2.5
2
1.5
1
0.5
0
